# Differences in cutaneous melanoma treatment and patient satisfaction

**DOI:** 10.1371/journal.pone.0205517

**Published:** 2018-10-25

**Authors:** Jakob D. Wikstrom, Lena Lundeberg, Margareta Frohm-Nilsson, Ada Girnita

**Affiliations:** 1 Dermatology and Venereology Unit, Department of Medicine Solna, Karolinska University Hospital, Karolinska Institutet, Stockholm, Sweden; 2 Department of Oncology and Pathology, Cancer Centre Karolinska, Karolinska Institutet, Karolinska University Hospital, Stockholm, Sweden; Northwestern University Feinberg School of Medicine Galter Health Sciences Library, UNITED STATES

## Abstract

Although clinical guidelines exist, the management of patients with cutaneous melanoma (CM) is a complex process that may vary between different care providers with potential dysfunctions ultimately mirrored in the overall patient satisfaction. The aim of the present study was to investigate the CM management as related to lead times, surgical quality and diagnosis communication with the hypothesis that the care may differ between providers and disparities may impact patient satisfaction. Medical records of 181 patients were retrospectively analyzed with parallel patient satisfaction evaluation by telephone interviews. Overall mean lead times from initial diagnosis until completion of all surgery and histopathology reports were 80–100 days and delays occurred at every step of the process. General practitioners performed excision biopsies faster however this was mitigated by slower histopathology processing. University level CM care showed less lag time between excision biopsy, wide local excision for thick melanomas and histopathology confirmation. University level care operated with twice the surgical margin as compared to general practitioners and non-university level specialists. Male patients had larger excision biopsy margins and significantly shorter lead times than female patients. Patient satisfaction rates were generally higher in the academic hospitals as compared to general practitioners and non-university dermatology clinics. Surprisingly, there was no correlation between lead times and patient satisfaction. Taken together, CM show substantial variation and caution should be practiced when using patient satisfaction as a quality indicator.

## Introduction

Health care organizations are transforming into value-based systems where not only quantity, but also quality of production is measured in order to improve medical services and make them more cost efficient [1]. Therefore it is crucial to identify critical and quantifiable key diagnoses whose incidence is high enough to assess their care quality. This is especially challenging in dermatology which comprises thousands of different diseases. Many dermatological conditions are non-lethal and cannot be analyzed by mortality outcomes or are too rare to have validated morbidity scores. However, skin cancers in general, and cutaneous melanoma (CM) in particular, are common and thus suitable for quality measurements. Within this group, thick CM with high risk for metastasis is suitable for evaluation by standard mortality and morbidity numbers. Thin CM on the other hand carries low morbidity and mortality; therefore other parameters than metastasis rate and death are needed for quality assurance evaluation.

In this study, we analyzed the current state of CM care in the Stockholm healthcare region by quantifying lead times, surgical quality, diagnosis communication and their impact on overall patient satisfaction.

## Methods

As the overall aim of the study was to examine CM management including patient satisfaction we obtained data both from medical records and telephone interviews as detailed below.

### Participants and study design

The study was largely designed as a retrospective cross-sectional study of all patients attending standard melanoma follow-up visits at the Karolinska University Hospital skin cancer clinic during the whole year of 2014. Follow-up visits typically occur 6–8 weeks after the surgery is completed. All patients diagnosed with *in situ* or invasive melanoma (n = 181), were included and all patients were above 18 years. Due to lack of proper medical records or not being able to reach patients by telephone the number of patients included in different categories (e.g. lead time from first visit to excision biopsy or patient satisfaction) varied from 141–172. In addition to the cross-sectional medical records based design we also interviewed patients by telephone regarding their satisfaction.

### Data collection

Data was collected both from the public hospitals computerized medical records system (Take Care) and from copies of private clinics´ medical records. Based on the type of intervention performed, we stratified the patient flows into the following groups:

1. General practitioners that performed primary excision biopsy (n = 32).
2. General practitioners and others that referred patients for primary excision biopsy to private dermatology clinics or private surgery clinics or a county hospital dermatology clinic (Danderyds hospital) (n = 111).
3. Any clinic that referred patients directly to Karolinska University Hospital dermatology clinic’s skin cancer unit including Karolinska University Hospital plastic surgery clinic (n = 38).

**Fig 1:**
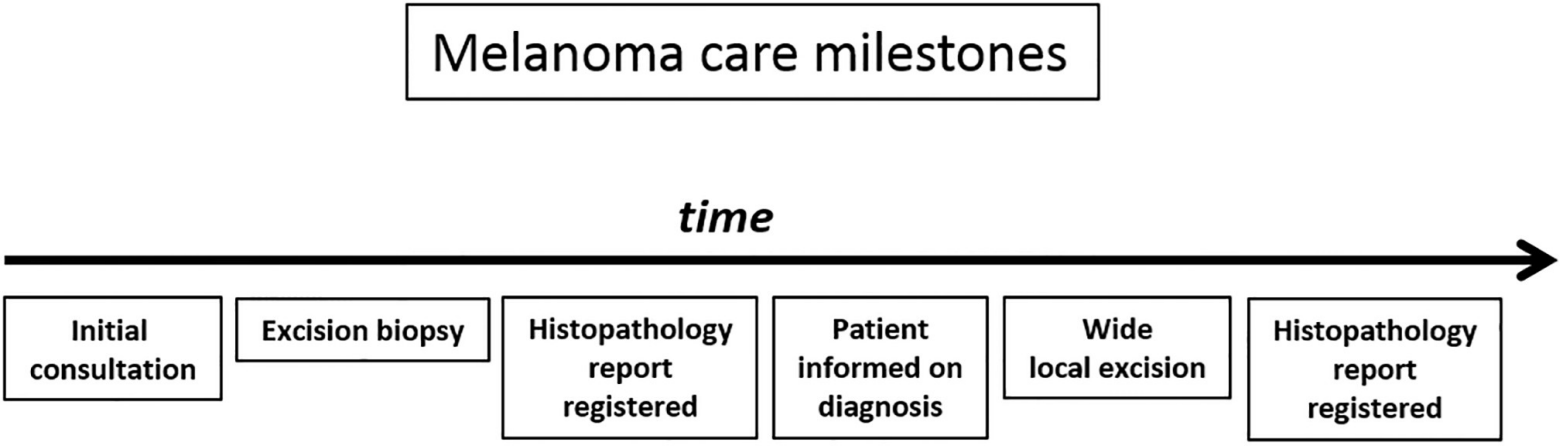
Melanoma care milestones. Boxes indicate information readily extracted from medical records.

**Fig 2:**
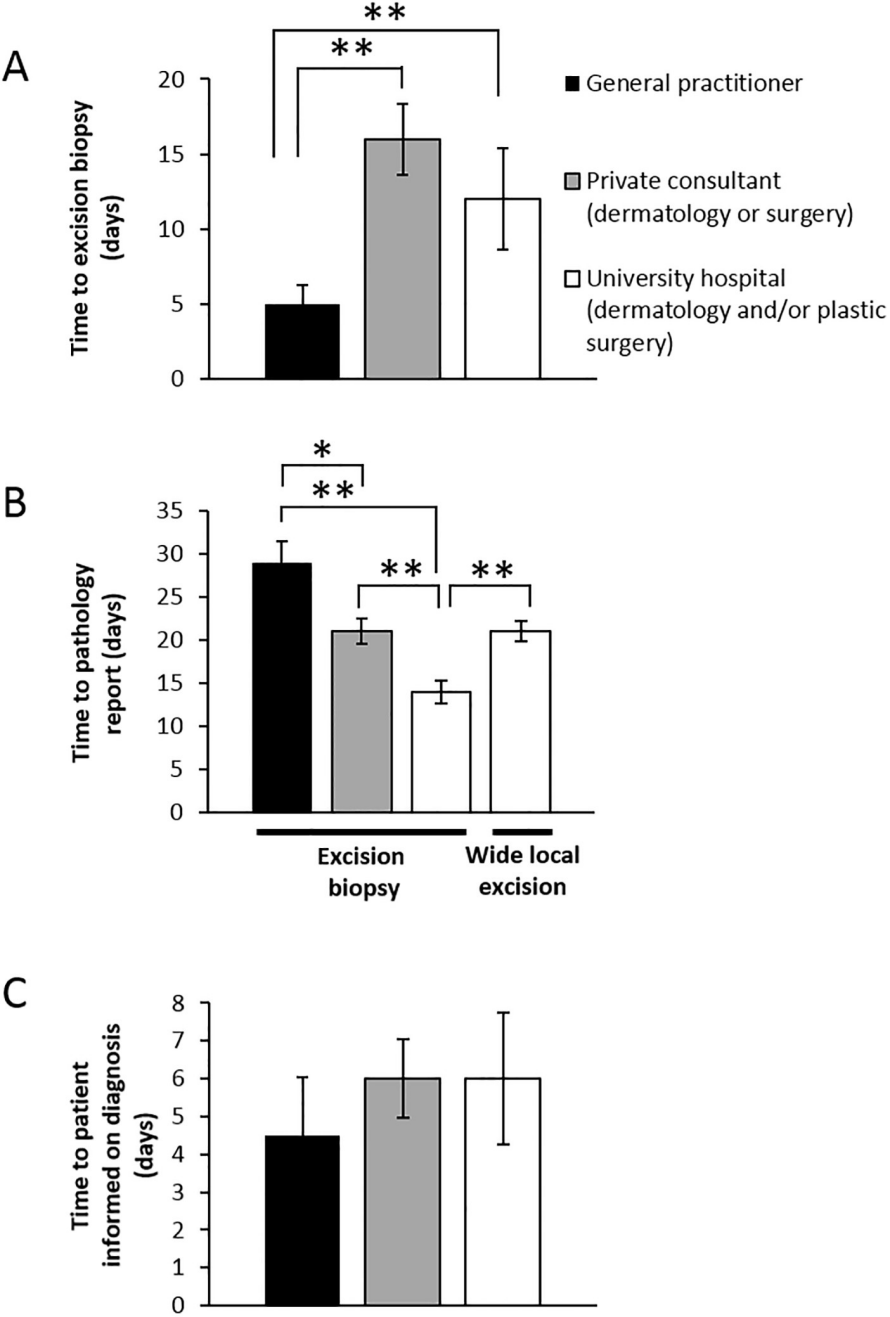
General practitioners excise melanomas faster however have slower histopathology assessment. A) Time from initial consultation until excision biopsy. B) Time from excision biopsy until histopathology report registered in medical records. C) Time from histopathology report registered until patient informed. Note that there were no significant differences between care levels in informing patients on melanoma diagnosis. *p<0.05, **p<0.01.

**Fig 3:**
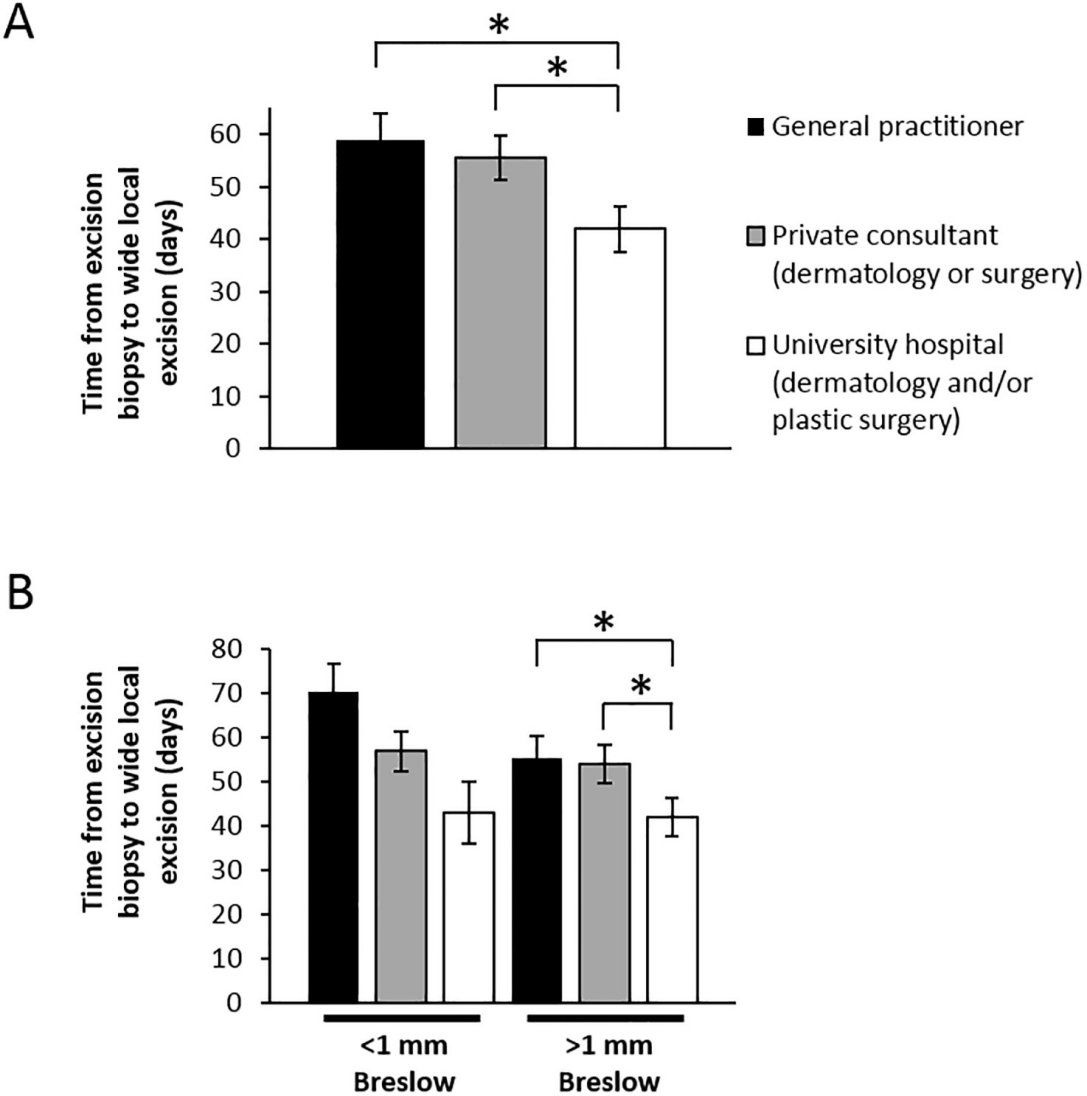
University level melanoma care show less inter-surgical delay. A) Time from excision biopsy until wide local excision regardless of Breslow tumor thickness. B) Time from excision biopsy until wide local excision stratified according to Breslow tumor thickness. *p<0.05.

**Fig 4:**
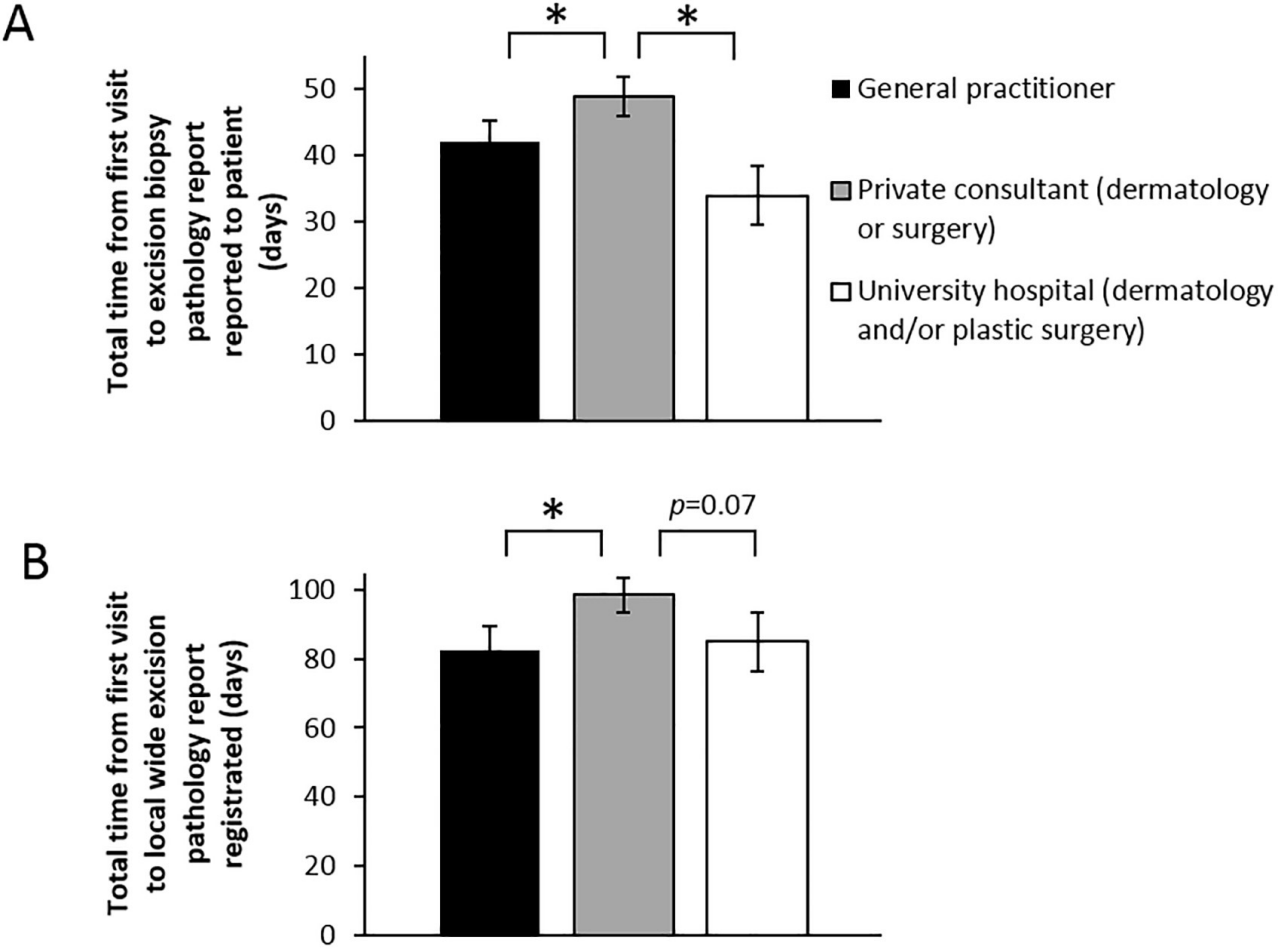
Private melanoma care show higher compiled delay. A) Time from initial consultation until patient informed on excision biopsy histopathology report (melanoma diagnosis). B) Total time from initial consultation until wide local excision histopathology report registered in medical records. *p<0.05.

Breslow tumor thickness, surgical margins as well as quantified lead times at clinical milestones and methods of communicating diagnosis (visit, phone call or letter) (Fig 1) were collected for each patient. Lead times were calculated from the first initial physician contact until the wide local excision histopathology reports were registered (Figs 1–4). Compiled lead times were calculated as such:

1. From initial consultation until the patient was informed about the melanoma diagnosis.
2. From initial consultation until the final wide local excision histopathology report was registered; both with the rational that these parameters provide a good overview of care and are assumed to be important for the patient.

As the histopathology report from the wide local excision is most often negative and given to patients at the follow-up visits several weeks after all surgery is completed; we did not analyze lead time to patients were informed on the final histopathology results.

Surgical quality was analyzed from excision biopsy histopathology reports.

In addition, patients were contacted over the phone in order to assess patient overall satisfaction of the melanoma care using a standard scale of 1–5, communicated to the patient as 1 = poor, 2 = fair, 3 = good, 4 = very good and 5 = excellent as previously published [2]. Sub group analysis based on age and gender was also performed.

### Ethics

Swedish Healthcare is required by law to systematically evaluate the quality of care given (SOSFS 2011:9, Swedish Medical Board’s statues). Ethical permission nor informed consent was not needed for this study as it was considered a quality of care follow-up and was subsequently waived by the clinic´s local ethics committee. Data was extracted from medical records and analyzed in an anonymous way from the quality control database. To avoid stressful situations, families of deceased patients were not contacted.

### Statistics

All variables presented were found normally distributed by Kolmogorov-Smirnov distribution fitting test and thus suitable for Students t-tests. T-tests were two-tailed, unpaired and with unequal variance and applied on figures in which p-values are indicated. Coefficient of determination (R^2^) was calculated by the Pearson formula. Data were analyzed by Excel (Microsoft, Redmond, WA) with XLStat macro (Addinsoft, New York, NY). Mean values are shown in figures except where otherwise indicated and error bars show standard error. P-values <0.05 were considered significant.

## Results

### Lead times between milestones

Basic patient characteristics are given in Table 1 and milestones showing the time points where lead time data was extracted from medical records are shown in Fig 1. Regarding time to excision biopsy, there was a significant difference between general practitioners and others lead times (Fig 2A). General practitioners often operated on patients immediately or with a short delay with a median waiting time between initial consultations to excision biopsy of only 5 days as compared to 16 and 12 days in private dermatology/surgery and university clinics respectively. On the other hand the lead times for the histopathology reports were the longest for the general practitioners. They had a median lead time of 29 days from excision biopsy until the report was registered in medical records. There were about 25% shorter lead times for the histopathology report registration in private dermatology/surgery clinics (median 21 days) while the lead times in university level care was considerably shorter (14 days) (Fig 2B). Wide local excision was only performed at university hospital level with a median lead time to registration of histopathology report of 21 days, significantly slower than for the same laboratory’s analysis of excision biopsies (Fig 2B). The median lead times from when histopathology reports were registered in medical records until the melanoma diagnosis was reported to the patient ranged from 4.5 to 6 days with no statistical significant differences between the different care levels (Fig 2C).

**Table 1:** Patient characteristics and initial diagnosis.

**Race (%White)**	100
**Male (%)**	61
**Age (mean, years):**	
General practitioner	66
Private consultant	68
University hospital	71
(no significant differences)	
**Excision biopsy (% of total)**	
General practitioner	18
Private consultant	60
University hospital	22
**Melanomas < 1mm (% of total)**	59

Lead times between excision biopsy and wide local excision was quantified for all melanomas. University level care showed significantly less delay in performing curative surgical procedures for the patients previously receiving excision biopsy at the same hospital. The median lead time was in this case 42 days as compared to 56 and 54 days for the patients referred by the general practitioners and private care respectively (Fig 3A and 3B), This is indicative of referral delay between primary care and university level care.

### Compiled lead times

Compiled median lead time A until patients were informed of melanoma diagnosis was significantly longer in private dermatology/surgery clinics (49 days vs. 42 days at general practitioners and 34 days at university level care) (Fig 4A). A similar pattern was found in compiled lead times B for wide local excision histopathology report registration in which private dermatology/surgery care showed a median of 99 days (Fig 4B). Swedish guidelines recommend a follow-up examination 6–8 weeks after surgery is completed [3] and thus we analyzed lead times at the Karolinska University Hospital dermatology clinic and found a median interval of 8.1 weeks (57 days).

### Diagnosis communication

**Fig 5:**
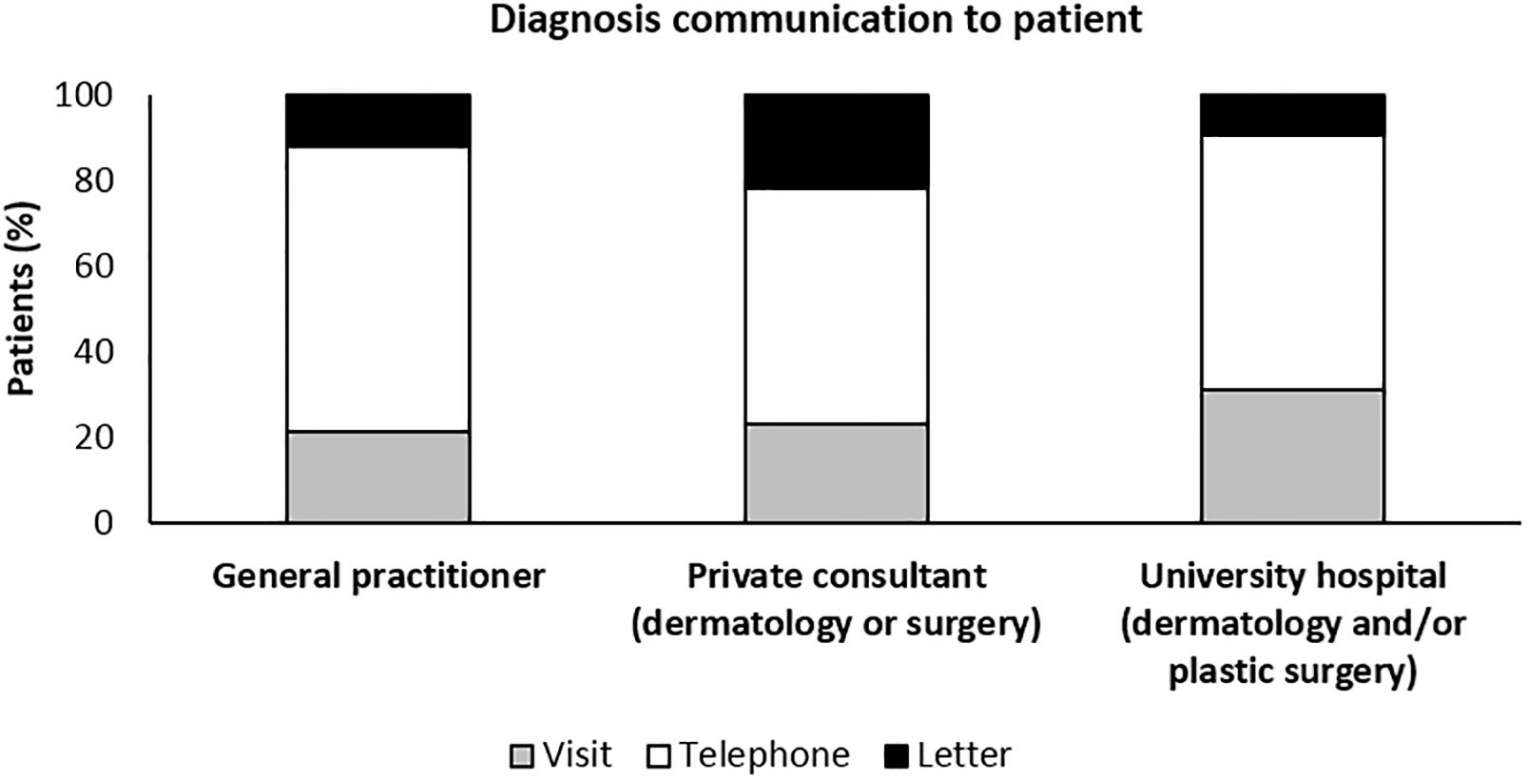
The majority of melanoma patients at all care levels are given diagnosis via telephone. Method of communicating melanoma diagnosis to patients.

Swedish guidelines recommend melanoma diagnosis to be given in person during a follow up visit if not otherwise previously decided [3]. By quantifying the method of communication, we found that telephone was the most common (55–66%), whereas 22–31% were informed in person and 9–22% via letter (Fig 5). Of note, patients informed via letter were usually hard to reach by other means or explicitly expressed their wish to be contacted that way.

### Surgical quality

**Fig 6:**
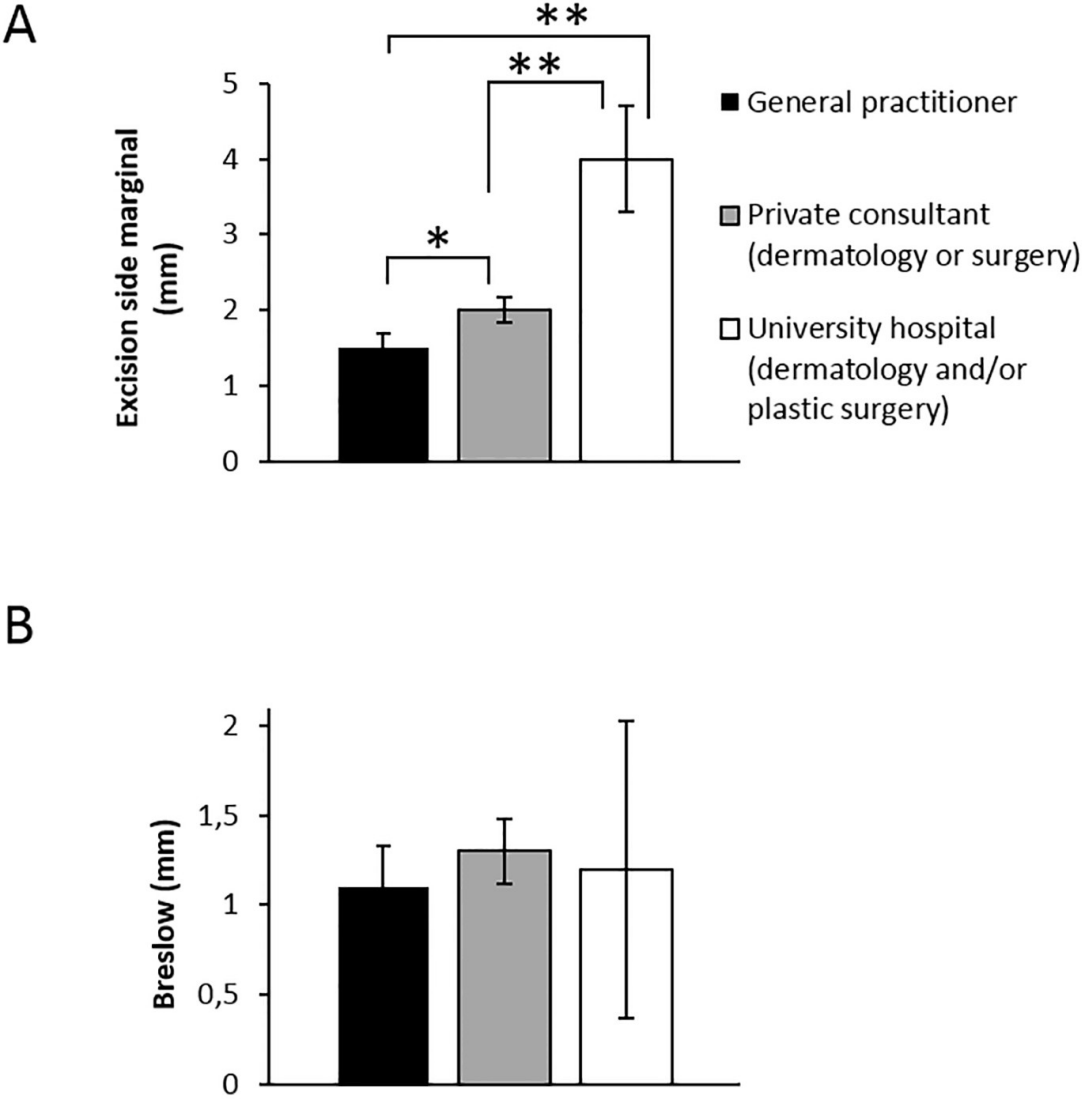
General practitioners excise melanomas with less margin despite similar Breslow thicknesses to other care levels. A) Excision side margins. B) Breslow thickness. Both A-B quantified from histopathology reports. *p<0.05, **p<0.01.

According to Swedish and international guidelines there are recommendations about the adequate excision side margins depending on the thickness of the melanoma [4] and therefore surgical quality was analyzed. No differences were found in Breslow thickness, however excision side margin was significantly smaller in the group of biopsies performed at general practitioners or private dermatology/surgery clinics as compared with the university level dermatology/plastic surgery group (Fig 6A and 6B; 1.5 mm and 2.0 mm vs. 4.0 mm). Excision biopsies were not radical or < 1mm in 14 (14%) (general practitioners), 15 (21%) (private dermatology/surgery) and 9 (15%) (university level) of cases. Overall excision biopsies were most commonly performed at private dermatology/surgery clinics and the majority of excision biopsies showed < 1mm Breslow thickness (Table 1).

### Gender differences

Previous studies report on gender differences in melanoma care and outcome [5]. We found a significant increase in compiled lead time B and significantly smaller surgical margins in female patients. There were no significant gender difference in Breslow thickness or frequency of melanoma *in situ* (Table 2).

**Table 2:** Patient gender analysis.

Total time (days, excision biopsy to wide local excision histopathology report registered)		
Male	85	*p* = 0.005
Female	103	
**Mis (% of total):**		
Male	19	
Female	18	
**Breslow (mm):**		
Male	1,3	*p*>0.005
Female	1,2	
**Surgical margin (mm)**		
Male	2	*p* = 0.015
Female	1,5	

### Patient satisfaction

**Fig 7:**
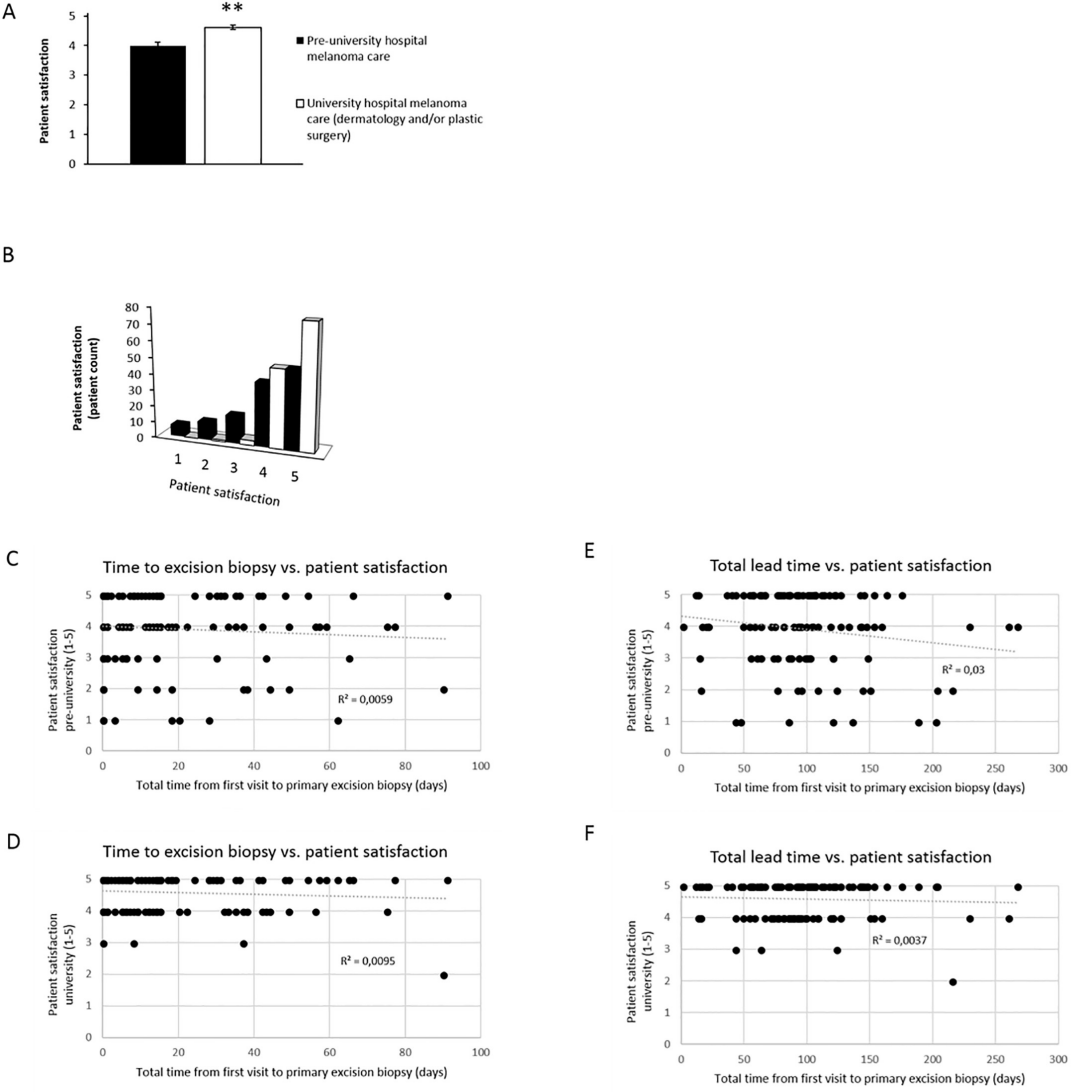
Patients are more satisfied with university care. Scale of 1–5 communicated to the patient by phone interview as 1 = poor, 2 = fair, 3 = good, 4 = very good and 5 = excellent. A) Median values. **p<0.01. B) Patient count stratified per grade Note that pre-university includes both general practitioners and private dermatology/surgery clinics. C-D) Lead time to excision biopsy from first consultation vs. patient satisfaction. Pre-university care (C), university-care (D). E-F) Total lead time from first consultation to wide local excision histopathology report registered vs. patient satisfaction. Pre-university care (E), university-care (F). Note that correlation coefficients (R^2^) are close to zero indicating lack of associations.

The patient satisfaction is often considered as an important parameter. The overall patient satisfaction was significantly higher for the group treated at university level (Fig 7A). As shown in Fig 7 patients were more satisfied with university level care (Fig 7A) with zero low grades (1–2) given for the university level care (Fig 7B). We hypothesized that lead times to excision biopsy and total lead times may have an impact on patient satisfaction; however, such association was not validated by statistical analysis as the correlation coefficients for these two variables were close to zero (Fig 7C–7F).

## Discussion

In summary, we found large delays at several time points in CM care. At all care levels melanoma excisions were delayed far beyond the European guidelines [4] recommendation of 4–6 weeks. General practitioners were faster to excise suspected lesions; however, had slower handling by their pathology laboratories. While the University hospital pathology service had the shortest handling times, no care level followed the Swedish guideline of maximum one week delay for the histopathology report [3]. There were no significant differences in doctor´s delay to report melanoma diagnosis to patients. Private dermatology/surgery clinics had the longest compiled lead times. University clinic treated lesions were excised with a significantly wider margin while there were no differences in Breslow thickness which could explain wider margins. Gender analysis revealed significantly shorter compiled lead times and wider surgical margins for males while Breslow thickness was gender neutral. Overall, patients were significantly more satisfied with university clinic level care. Surprisingly, despite a large range of lead times, no correlation was found between compiled lead times and patient satisfaction.

### Treatment differences

#### Lead times

The state of melanoma care in Stockholm is unknown to a large degree except limited lead time, surgical and histopathology parameters reported from the national melanoma register [6]. Importantly, this register excludes the large portion of patients diagnosed with melanoma *in situ*. In 2014, the median lead time from first consultation visit until diagnosis was given to the patient was 37 days in Sweden as a whole and 38 days in Stockholm [7]. This is similar to this study where we found a median lead time of 34–49 days depending on care level (Fig 4A). The median lead times to excision biopsy and to histopathology report was 21–24 days and 24 days respectively in Stockholm in 2010 [8] while we found median lead times of 5–16 and 14–29 days respectively (Fig 2B). Thus, there may have been some reduction in lead times. Furthermore, the current Swedish guidelines on standardized cancer care recommend a maximum of 7 days until excision biopsy and 14 days until giving diagnosis to patient [3]. We conclude that only primary care performs excision biopsy within this time frame (Fig 2A) and that all care levels show significant delays in giving diagnosis (Fig 2B and 2C).

In comparison a study from Umeå in northern Sweden on 71 patients reported on a number of lead times including median delay to excision biopsy of 0–4.5 days (general practitioners vs. dermatology clinic) and median delay of 7–13 days for registration of the excision biopsy histopathology report into medical records [9]. The finding that general practitioners excise melanomas faster appears similar in our study. Further, a multi-national study where 50 Swedish patients from Southern Sweden were included showed an overall median diagnostic delay of 32 days [10]. This delay is similar to general practitioners in Stockholm but longer compared to private dermatology/surgery and university level care (Fig 2B). Moreover, a large recent American study showed delay to excision biopsy of more than 1.5 months in 20% of Medicare patients and less so in dermatology clinics [11], which was also the case in the Umeå study [9]. On the contrary, our study showed that the general practitioners in Stockholm show less delay to excision biopsy than secondary and tertiary care levels, perhaps due to higher accessibility.

A variety of factors may lie behind the reported lead times and the differences between primary, secondary and tertiary care. First, patient overload that prohibits the physician to perform an immediate excision biopsy is likely to be a problem at all levels. Second, histopathology turnover times are long, at least in part due to Swedish pathologist shortage. Third, doctor’s delay is likely to be important as the doctor performing the excision is normally the only one to whom the histopathology report is addressed. Thus, if that doctor is absent, communication of a melanoma diagnosis to the patient may be delayed as well as referral to wide local excision. Fourth, the fact that the healthcare in Stockholm is fragmented may contribute. Patients commonly first attend general practitioners that refer to private dermatology, which in turn refer to the university dermatology or plastic surgery clinics for wide local excision and follow-up. To circumvent some of these obstacles, simple fast track referral systems that enable rapid access to dermatologists specialized in melanoma may be a solution. A recent study showed that this model improves melanoma prognosis and increases the fraction of tumors diagnosed as melanoma *in situ* [12]. Also, reimbursement systems could also be set to reduce payments if lead times are too long. Moreover, optimally, all clinics should use the same computerized medical records to enable easy continuous quality monitoring.

Lead times may be of importance as melanoma is fast growing and prognosis strictly dependent on tumor thickness (Breslow). CM is also associated with high levels of anxiety [13], which extended lead times may unnecessarily prolong. Further, healthcare cues carry high costs [14]. Despite these considerations international current guidelines provide few lead time recommendations. American guidelines give no lead time recommendation at all [15], while European guidelines recommend excision biopsy within 4–6 weeks [4]. Swedish guidelines recommend excision biopsy to be performed within 7 days, diagnosis given to patient within 14 days and sentinel node biopsy to be performed within 12 days [16] while a dermatology follow-up visit 6 weeks after all surgery is completed is recommended [17]. A UK guideline recommends that melanoma suspected lesions should be referred to specialty clinics within two weeks, however makes no recommendation on surgical lead time [18].

Extended lead times may have several consequences. First, as melanoma is a fast growing tumor, significant diagnostic delays will most certainly worsen outcome, e.g. 12–18 months delay in acral and subungal melanoma [19]. However the effect of shorter delays in the order of weeks-months may not be important as a retrospective UK study found that the time interval between excision biopsy and subsequent wide local excision had no effect on survival, disease-free survival or recurrence rates [20]. Second, shorter lead times may be more cost effective [21]. As melanoma is a potentially deadly diagnosis where time does matter and delays cause anxiety and increased costs, it can readily be argued that lead time should be considerably decreased. The authors believe that a total of 4 weeks from diagnosis to final pathology report is reasonable and achievable as compared to the current lead time of 3 months, as observed in this study.

#### Surgical quality

American and European guidelines recommend excision biopsy margins of 1–3 mm, while Swedish guidelines recommend excision biopsy margins of 2–5 mm [15, 17, 22]. A few studies suggest that surgical quality may not matter as incomplete excision biopsy does likely not affect melanoma metastasis potential [23] and does not affect histopathological assessment to any large degree [24] or alter surgical management [25]. However, a large Cochrane review concluded that current randomized trial evidence is insufficient to address optimal excision margins for primary cutaneous melanoma [26]. Thus, it cannot be excluded that excision margins may matter for survival.

Surgical margins may be underestimated on fixed biopsy material, however this underestimation is expected to be relatively minor as mean post excision skin shrinkage is only 12% (width) or 21% (length) [27]. We found a substantial number of cases that were excised with narrow or no margin as well as a clear difference in margins between non-university *vs*. university-level clinics. As it is not clear if incomplete excision jeopardizes outcome [23] we believe caution should be practiced. Moreover, surgical quality may serve as an easily quantifiable surgical quality marker.

#### Diagnosis communication

Previous studies have reported dissatisfaction among cancer patients in how diagnosis was delivered [28]. Swedish and other guidelines recommend that melanoma or any other cancer diagnosis is given in person [17]. Yet, one study did not find any difference in patient satisfaction or anxiety between in person and telephone communication of cancer diagnosis [29]. Somewhat surprisingly, we found that the majority of patients are given CM diagnosis over the phone and some even by letter (Fig 5). This is in comparison with an American study where 82% of patients were given diagnosis in person, either in an outpatient or hospital setting [30]. Not surprisingly patients were more satisfied by having the diagnosis disclosed in person [30].

#### Gender inequality

Men show worse melanoma survival than women independent of tumor stage, a phenomenon that is incompletely understood [31]. To our best knowledge no studies to date have examined gender disparities regarding CM diagnostic delay [5]. We found that male patients have 18% shorter total lead time and their tumors were excised with 33% wider margins (Table 2). The reason for this is not clear as Breslow thickness did not differ between male and female patients. While extended lead times of a couple of weeks may extend patient anxiety unnecessarily, it is unlikely that prognosis is affected [20].

#### Patient satisfaction

One outcome often disregarded is patient satisfaction. It was previously reported that patients that underwent excision biopsies at dermatologists were more satisfied and delay in diagnosis increased the likelihood of dissatisfaction [32]. Waiting for cancer diagnosis results may also cause stress. Our data show higher satisfaction among university level dermatology/plastic surgery treated patients, however, surprisingly, we found no correlation between delay and satisfaction as was previously reported [32]. A topic for future studies may be if patient satisfaction is influenced by patient information level.

#### Conclusions

The overall finding is that the melanoma care is characterized by extended delays and high variability in surgical quality among care givers. The impact of delays and surgical variability on melanoma outcome is yet to be investigated; however, it is reasonable to believe that at least some of the delays could be avoided by a more efficient system. Despite delays patient satisfaction was overall high and surprisingly not correlated to lead time albeit with higher satisfaction associated with university hospital treated patients group.

## Supporting information

S1 Dataset (XLSX)
