# Correction: Differences in cutaneous melanoma treatment and patient satisfaction

**DOI:** 10.1371/journal.pone.0246145

**Published:** 2021-01-22

**Authors:** Jakob D. Wikstrom, Lena Lundeberg, Margareta Frohm-Nilsson, Ada Girnita

The first, third, and fourth authors, Jakob D. Wikstrom, Margareta Frohm-Nilsson, and Ada Girnita, should be noted as contributing equally to this work.
